# Age differences in option choice: Is the option framing effect observed among older adults?

**DOI:** 10.3389/fpsyg.2022.998577

**Published:** 2022-09-29

**Authors:** Kouhei Masumoto, Min Tian, Kenta Yamamoto

**Affiliations:** Graduate School of Human Development and Environment, Kobe University, Kobe, Japan

**Keywords:** cognitive bias, decision-making, option framing, motivation, older adult

## Abstract

Previous studies reported that consumers choose a higher number of options in subtractive framing (-OF), which delete the unnecessary options from the full model with all options chosen than in additive framing (+OF), which adds options to a simple base model. The purposes of this study are to examine the effect of age on option framing and the differences of product type on the option framing effect using two product scenarios (travel package and medical examination). Participants were 40 younger and 40 older adults. We measured the number of options chosen, total price, choice difficulty, and choice satisfaction. In addition, cognitive functions (coding, symbol search, digit span, arithmetic, and information) were assessed. Results revealed that older and younger adults chose more options in the -OF condition for both the scenarios. For the medical examination, older adults chose more options than did the younger adults in both -OF and +OF conditions. Developmental shift in goals and motivation related to life-span may explain the differences between the age differences.

## Introduction

At present, many countries are experiencing rapid population aging. As a result, older households that comprise older couples or older adults living alone, are increasing. This implies an increasing number of situations in which older adults will have to make important choices for themselves. Recently, there has been an increase in the number of products from which consumers can choose options that meet their various demands, such as special provisions in insurance and customization of automobiles and computers. This study seeks to determine whether the decision-making process for choosing such options changes with age?

Option framing is used to examine consumers’ decisions in terms of whether to choose or reject an option (Park et al., 2000). There are two types of option framing (Park et al., 2000): additive framing, which adds options to a simple base model (hereafter + OF), and subtractive framing, which removes undesired options from a full model in which all options are selected (hereafter –OF). Previous studies report an option framing effect, showing that consumers choose more options in the –OF condition than in + OF. This effect has been confirmed with a variety of products and services, such as automobiles (Park et al., 2000; Biswas and Grau, 2008; Biswas, 2009; Park and Kim, 2012; Cheng et al., 2013; Herrmann et al., 2013; Lu and Jen, 2016; Peng et al., 2016), computers (Park et al., 2000), condominium (Pornpitakpan, 2009, 2010), pizzas (Levin et al., 2002; Cheng et al., 2013), travel packages (Jin et al., 2012; Lu and Jen, 2016; Chen, 2019), and treadmills (Park et al., 2000).

Previous studies explain the occurrence of the option framing effect based on “loss aversion,” “*status quo*,” “endowment effect,” and “difference between choosing and rejecting” (Levin et al., 2002; Biswas and Grau, 2008; Jin et al., 2012; Park and Kim, 2012; Herrmann et al., 2013).

The reference point is different for –OF and + OF. In the former, the reference point is the full model, from which the consumer removes undesired options. Therefore, the consumer is conflicted between the loss of functionality and the financial gain. In the latter condition, the reference point is the base model, and the consumer is conflicted between the gain in functionality and the monetary loss. People overestimate losses more than they do gains (Kahneman and Tversky, 1979). Hence, in the –OF, consumers remove fewer options to avoid functional loss, while in the + OF, they add fewer options to avoid monetary loss (Park and Kim, 2012; Herrmann et al., 2013).

In addition, the choice of an option involves a trade-off between high functionality and low price. In the case of uncertain and difficult choices, the choice not to act and change the *status quo* is easier to justify than the choice to act and change the *status quo*; moreover, the anticipated regret about the results is smaller (Ritov and Baron, 1992). Therefore, the *status quo* is maintained by not adding an option in the + OF and by not removing an option in the –OF, and the option framing effect is observed (Park and Kim, 2012; Herrmann et al., 2013).

Option framing is also interpreted by the endowment effect (Levin et al., 2002; Jin et al., 2012), which contends that people place greater value on things they own than on things they do not own (Kahneman et al., 1990). Levin et al. (2002) descried that merely considering the prospect of having that thing increases its value, even if people do not actually own it. In the full model, the –OF, there is a “virtual” endowment effect, wherein consumers think they own all the options. Therefore, consumers are hesitant to remove options in this condition.

Another interpretation is that the option framing effect is explained by the difference between choice and rejection (Park et al., 2000; Jin et al., 2012). Positive information is more important in choosing than in rejecting something. Conversely, negative information is more important in rejecting than in choosing something (Shafir et al., 1993). As most of the information given in product selection is positive, consumers have a reason to add an option, but not to remove one. As a result, consumers are expected to choose more options in the –OF than in the + OF condition (Park et al., 2000).

The primary purpose of this study was to examine the effect of age on the option framing effect. Most previous studies of the option framing focus mainly on the choices of younger adults. To the best of our knowledge, there was only one study targeting older adults. Peng et al. (2016) report that older adults showed an option framing effect, choosing more options and accepting higher prices than younger and middle-aged adults. Although they used the option choice of automobiles as a task, in this study, we examined the effect of option framing using two different products (travel package and medical examination).

In terms of the effect of age on option framing, H1a, H1b, and H1c can be formulated. These hypotheses are based on cognitive decline with age, compensation through experience and knowledge, and motivation for choice. This study examines which of these hypotheses are valid or if none of them are.

It is well known that cognitive function declines with age. As for cognitive processing in option framing, Biswas (2009) reports that, when making rational choices, decisions are based on deliberate reasoning and are less affected by option framing. However, when making heuristic, experiential choices, decisions are made automatically and are more susceptible to framing effects. Option framing involves a trade-off between higher quality and lower price. Because such conflicts involve cognitive load and negative emotions, the increasing difficulty of the trade-off perpetuates the *status quo* (Luce et al., 1999). Working memory and executive functions, which are the basis of deliberative processes, decline with age (Park et al., 2002; Zelazo et al., 2004). Implicit cognitive processing, however, which is related to intuitive and experiential processes, is less affected by aging (Fleischman et al., 2004; Horton et al., 2008). The age-related decline in deliberative processes predict that older adults will experience decision bias related to heuristic processing, especially in unfamiliar situations (Peters et al., 2007). Therefore, in option choice, where trade-offs that require cognitive effort occur, older adults are likely to be more strongly affected by option framing than younger adults, as they tend to make more heuristic judgments. It has been reported that –OF are more difficult than + OF (Park et al., 2000) because individuals tend to value functional loss more than monetary loss (Hardie et al., 1993), and are more used to choosing options, rather than removing them (Shafir et al., 1993). In this study, the choice difficulty is also expected to be higher for –OF than + OF; therefore, older adults with cognitive decline will choose more options in –OF.

**H1a**: Older adults choose fewer options in the + OF and more options in the –OF, compared to younger adults.

However, previous research shows that in decision making, the decline in fluid intelligence—such as processing speed and working memory—due to aging can be compensated for by crystallized intelligence, such as previous experience and knowledge (Peters et al., 2007; Li et al., 2013). Peters et al. (2007) point out the possibility of older adults being able to develop useful knowledge for decision making through life experiences, such as shopping, and that such experiences can help avoid biases in decisions. A study by Peng et al. (2016) reports that age did not affect the option framing effect. They suggest that the rich experience of older adults compensates for age-related declines in cognitive function. Therefore, the following results are also predicted.

**H1b**: There is no difference in the option framing effect between older and younger adults.

Furthermore, differences in the goals of choice between older and younger adults may affect option framing. Socio-emotional selectivity theory (Carstensen et al., 1999; Carstensen, 2006) states that older adults who perceive their time as limited tend to attach greater importance to emotional goals. Emotionally meaningful things and events are valued highly, and they tend to invest their cognitive and social resources into obtaining emotional value. In support of this theory, studies report that older adults pay more attention to and are more likely to remember positive than negative information and make behavioral choices that favor positive information (Fung and Carstensen, 2003; Mather and Carstensen, 2005; Notthoff and Carstensen, 2014; Masumoto et al., 2020). Meanwhile, younger adults tend to pay more attention to negative information to avoid losses (Baumeister et al., 2001). As mentioned earlier, based on the reference point, positive information for –OF differs from that of + OF. In –OF, the positive aspect of removing an option is monetary gain, while the negative aspect is functional loss. In + OF, adding an option enhances functionality, while the negative aspect is monetary loss. Therefore, if older adults value positive information and younger adults value negative information, then in –OF, older adults value monetary gain. Therefore, older adults will choose fewer options, compared to younger adults, who are attempting to avoid functional losses. In + OF, older adults value functional gain, while younger adults value avoiding monetary loss. Therefore, older adults will choose more options than younger adults. In other words, contrary to H1a, the following hypothesis is proposed.

**H1c**: Older adults choose fewer options in –OF and more options in + OF, compared to younger adults. Therefore, the option framing effect will not be observed or will be smaller than for younger adults.

The second purpose of this study is to examine differences in option framing effects across products. We use two types of products, a travel package and a medical examination, and expect the effects of aging on choice to be different in each. The goals of these two option choices are different. Regarding the travel package, we choose an option to have fun, while for a medical examination, we choose an option to avoid a bad situation. Older adults only engage with both positive and negative information when avoiding negative information would have a detrimental effect, as in health-related decision making (Reed and Carstensen, 2012). Baltes (1997) mentions that life span changes according to the allocation of resources (e.g., material, technological, social, economic, or psychological) to functions of development as follows: in childhood, the primary allocation is directed toward growth; in adulthood, resources are devoted to maintenance and resilience; and in old age, several resources are allocated toward regulation and management of loss. Subsequent studies confirm this shift in goals (Ebner et al., 2006). With a focus on the management of loss, positive information in both -OF and + OF will increase examination options more than monetary loss. Therefore, we hypothesize:

**H2a**: For the medical examination, although younger adults show an option framing effect, older adults choose more options to manage losses due to disease, and there is no difference in option choice between + OF and –OF. For the travel package, one of the above hypotheses H1a, b, or c is expected to hold, because this option choice is less related with growth, maintenance, and loss management.

Participants in this study were also required to report their choice satisfaction after choosing the options. Older adults, who tend to focus on positive information, report greater satisfaction after making a decision (English and Carstensen, 2015). If older adults value emotional satisfaction more than younger adults (Carstensen, 2006), the following hypothesis is proposed for choice satisfaction.

**H2b:** Regardless of products, older adults are more satisfied with their choice than younger adults in both –OF and + OF.

## Materials and methods

### Experimental design

This study adopted a 2 (product: travel package vs. medical examination) × 2 (age group: younger vs. older) × 2 (option framing: + OF vs. –OF) study design, with product as a within-participant variable and age group and option framing as between-participant variables.

### Participants

The participants included 40 younger adults (15 male and 25 female) between 18 and 26 years of age (mean = 21.03, SD = 1.75) and 40 healthy older adults (16 male and 24 female) between 63 and 86 years of age (mean = 72.33, SD = 5.05). All older adults were registrants of the Silver Human Resource Association, and all younger adults were recruited from among the students at the author’s affiliate university. Older adults were paid 4,000 Yen (including transportation costs) and younger adults were paid 2,000 Yen as compensation at the end of the experiment. All the participants verbally reported that they did not have any history of neurological disorders or psychiatric illnesses. Informed consent for participation was obtained from all participants.

Half the participants in each age group were assigned to + OF and the other half to –OF. In the + OF, the mean age of the older adults (male = 8, female = 12) was 72.40 (SD = 4.71) and that of the younger adults (male = 9, female = 11) was 20.80 (SD = 1.44). In the –OF, the mean age of the older adults (male = 8, female = 12) was 72.25 (SD = 5.50) and that of the younger adults (male = 6, female = 14) was 21.25 (SD = 2.02).

Option framing is a robust effect (Biswas, 2009; Pornpitakpan, 2009). We set the effect size to 0.4 and calculated the sample size required to obtain a power of 0.8 at α = 0.05 using G*power (Faul et al., 2007). The required number of participants was shown to be 56 (14 in each cell). As a total of 80 younger and older adults participated in this study (20 in each cell), the sample size was sufficient to detect option framing effects.

To measure the cognitive function of the participants, we used several subtests (coding, symbol search, digit span, arithmetic, and information) of the Japanese version of the Wechsler Adult Intelligence Scale, Third Edition.

### Option framing task

The two products used in this study—the travel package and medical examination— (Appendix A, B) were both priced at 77,000 Yen for the basic model. The travel package was a domestic trip to Okinawa with friends, and participants were informed that the hotel and flight costs were included in the basic model. For the medical examination, participants were informed that the basic model included a medical interview, measurements of height, weight, vision, hearing, and blood pressure, a urinalysis, a chest x-ray, and a hematological examination. Both products comprised 10 options, and the total amount of the options was 135,500 Yen. In the + OF condition, a base model (basic price) and a list of options were presented, and participants were asked to add the options that they needed. In the –OF condition, a full model (total price) and a list of all checked options were presented, and participants were asked to delete the options that they did not need. Participants in each age group were randomly assigned to one of the two option framing conditions, + OF or –OF. In the experiment, the option names, option descriptions, prices, and checkboxes were presented on the monitors.

After selecting an option in each scenario, participants were asked to rate the choice difficulty (It was difficult to choose options) and satisfaction (I am satisfied with my present choice) on a 7-point scale ranging from 1 (*Strongly disagree*) to 7 (*Strongly agree*).

### Procedure

First, the experimenter explained the purpose of the experiment to the participants and obtained their written informed consent.

Next, the option framing task was conducted. Participants were asked to sit in front of the PC. As a practice trial, participants performed the commemorative album option choice scenario to ensure that they understood the task; next, the experimenter administered the information on the travel package and medical examination. The order in which the travel package and medical examination were administered was counterbalanced among the participants. To prevent participants from making choices without checking the options, they were asked to read aloud the name, description, and price of the option for each product. Then, in the option choice phase, they were instructed to select or remove the options as if they were actually purchasing the product.

After the completion of the option framing task, a break of about 15 minutes was allowed to test the cognitive function.

## Results

### Cognitive function

Table 1 presents the results of the cognitive tests (coding, symbol search, digit span, arithmetic, and information) for each age group by option framing. A two-way analysis of variance (ANOVA) with age group (young, old) and option framing (+ OF, –OF) as between-participant variables was conducted for each subtest. The results showed that the main effects of age group were significant for coding, symbol search, digit span, and arithmetic, while the main effects of condition and interaction were not significant: coding [the main effect of age group; *F* (1,76) = 98.00, *p* < 0.001, η^2^*_*p*_* = 0.56, the main effect of option framing; *F* (1,76) = 0.25, *p* = 0.62, η^2^*_*p*_* = 0.003, and the interaction; *F* (1,76) = 1.73, *p* = 0.19, η^2^*_*p*_* = 0.02], symbol search [the main effect of age group; *F* (1,76) = 129.08, *p* < 0.001, η^2^*_*p*_* = 0.63, the main effect of option framing; *F* (1,76) = 1.39, *p* = 0.24, η^2^*_*p*_* = 0.02 and the interaction; *F* (1,76) = 2.46, *p* = 0.12, η^2^*_*p*_* = 0.03], digit span [the main effect of age group; *F* (1,76) = 59.65, *p* < 0.001, η^2^*_*p*_* = 0.44, the main effect of option framing; *F* (1,76) = 0.24, *p* = 0.63, η^2^*_*p*_* = 0.003 and the interaction; *F* (1,76) = 0.06, *p* = 0.81, η^2^*_*p*_* = 0.001], and arithmetic [the main effect of age group; *F* (1,76) = 102.39, *p* < 0.001, η^2^*_*p*_* = 0.57, the main effect of option framing; *F* (1,76) = 0.09, *p* = 0.76, η^2^*_*p*_* = 0.001 and the interaction; *F* (1,76) = 0.21, *p* = 0.65, η^2^*_*p*_* = 0.003]. All main effects and interactions were not significant for information [the main effect of age group; *F* (1,76) = 0.18, *p* = 0.68, η^2^*_*p*_* = 0.002, the main effect of option framing; *F* (1,76) = 1.31, *p* = 0.26, η^2^*_*p*_* = 0.02 and the interaction; *F* (1,76) = 0.00, *p* = 1.00, η^2^*_*p*_* = 0.000].

**TABLE 1:** Cognitive function of the older and younger groups.

Cognitive function		Older		Younger	
		+OF	–OF	+OF	–OF
Speed of processing	Coding	71.25 (15.78)	73.75 (12.58)	105.55 (11.07)	100.00 (14.78)
	Symbol search	32.35 (5.44)	32.90 (5.45)	50.70 (6.25)	46.80 (7.93)
Working memory	Digit span	15.95 (3.98)	16.15 (3.76)	22.10 (3.75)	22.70 (3.16)
	Arithmetic	14.05 (3.27)	14.15 (3.51)	21.00 (2.03)	20.50 (2.72)
Verbal comprehension	Information	19.20 (3.74)	18.25 (5.08)	19.55 (2.89)	18.60 (2.72)

These results suggested that processing speed (coding and symbol search) and working memory (digit span and arithmetic) declined with age, while verbal knowledge (information) did not decline with age. This, in turn, indicated that older adults who participated in this study can be considered to have typical cognitive function. These functions were not significantly different between the option framing condition groups.

Additionally, before participating in the experiment, all the participants verbally reported that they did not suffer from dementia or neurological disorders. This was also confirmed by the fact that none of the participants had extremely low scores on any of the cognitive tests—which would suggest the presence of dementia.

### Option framing

**FIGURE 1:**
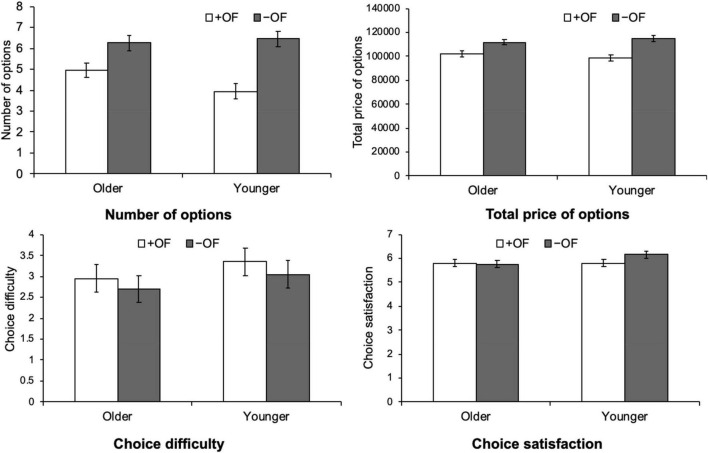
Age differences in the effects of framing on the number of options chosen, total price, choice difficulty, and choice satisfaction for the travel package.

**FIGURE 2:**
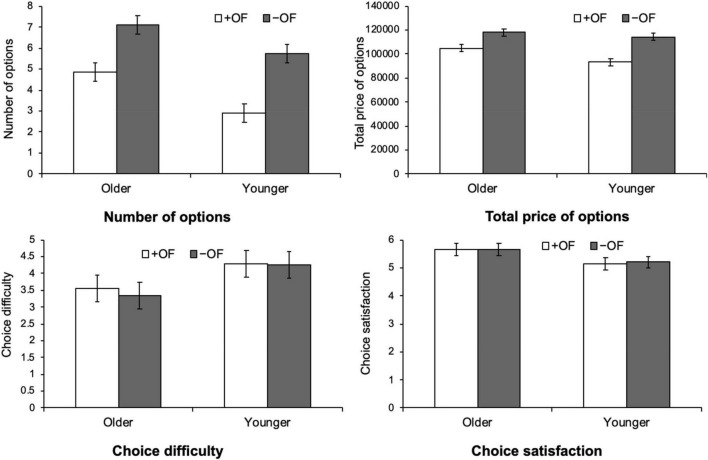
Age differences in the effects of framing on the number of options chosen, total price, choice difficulty, and choice satisfaction for the medical examination.

Figures 1, 2 show the results of the customization for each travel package and medical examination, respectively.

We conducted a three-way ANOVA for each variable—number of options chosen, total option price, choice difficulty, and satisfaction—with product (travel package, medical examination) as a within-participant variable, age group (young, old) and option framing (+ OF, –OF) as between-participant variables.

### Number of options chosen

In terms of the number of options chosen, there was a significant main effect of age group [*F* (1, 76) = 9.46, *p* = 0.003, η^2^*_*p*_* = 0.11] and option framing [*F* (1, 76) = 44.56, *p* < 0.001, η^2^*_*p*_* = 0.37]. The main effect of product was not significant [*F* (1,76) = 1.37, *p* = 0.25, η^2^*_*p*_* = 0.02]. These results indicate the option framing effect of choosing more options in –OF than in + OF, for both older and younger adults. In terms of the interaction, only the interaction between product and age group was significant [*F* (1,76) = 8.56, *p* = 0.005, η^2^*_*p*_* = 0.10]; the other interactions were not significant [product and option framing, *F* (1,76) = 2.31, *p* = 0.13, η^2^*_*p*_* = 0.03; age group and option framing, *F* (1,76) = 1.82, *p* = 0.18, η^2^*_*p*_* = 0.02; third order interaction, *F* (1,76) = 0.49, *p* = 0.49, η^2^*_*p*_* = 0.01]. As the interaction between product and age group was significant, we performed a *post hoc* analysis using Bonferroni’s multiple comparison test. The results showed that the older group chose significantly more options than the younger group for the medical examination (*p* < 0.001). As for travel packages, there was no significant difference between the age groups (*p* = 0.27). A comparison in the number of options chosen between products, by age group, showed that younger adults chose more options for travel package than for medical examination (*p* = 0.005). No significant differences were found between products for older adults (*p* = 0.22).

### Total price

With regard to the total option price, a three-way ANOVA showed that the main effect of the option was significant [*F* (1,76) = 43.42, *p* < 0.001, η^2^*_*p*_* = 0.36] and the total price was higher for –OF than + OF. There was no significant main effect of product [*F* (1,76) = 0.33, *p* = 0.57, η^2^*_*p*_* = 0.004] and age group [*F* (1,76) = 2.70, *p* = 0.11, η^2^*_*p*_* = 0.03]. For the interaction, only the interaction between product and age group was significant [*F* (1,76) = 6.23, *p* = 0.02, η^2^*_*p*_* = 0.08]; the other interactions were not significant [product and option framing, *F* (1,76) = 2.00, *p* = 0.16, η^2^*_*p*_* = 0.03; age group and option framing, *F* (1,76) = 2.21, *p* = 0.14, η^2^*_*p*_* = 0.03; third order interaction, *F* (1,76) = 0.10, *p* = 0.75, η^2^*_*p*_* = 0.001]. A *post hoc* analysis was conducted for the interaction between product and age group. Comparisons between age groups by product showed that the total prices were significantly higher for older adults than for younger adults, only in medical examinations (*p* = 0.01). Comparisons between products by age group showed that the total price of medical examinations was significantly higher than that of travel package (*p* = 0.03) only for older adults.

### Choice difficulty

In terms of choice difficulty, a three-way ANOVA showed a significant main effect of product [*F* (1,76) = 14.57, *p* < 0.001, η^2^*_*p*_* = 0.16] and age group [*F* (1,76) = 4.39, *p* = 0.04, η^2^*_*p*_* = 0.06]. There was no significant main effect of option framing [*F* (1,76) = 0.49, *p* = 0.49, η^2^*_*p*_* = 0.006] and no significant interactions [product and age group, *F* (1,76) = 1.02, *p* = 0.32, η^2^*_*p*_* = 0.01; product and option framing, *F* (1,76) = 0.11, *p* = 0.74, η^2^*_*p*_* = 0.001; age group and option framing, *F* (1,76) = 0.01, *p* = 0.93, η^2^*_*p*_* = 0.00, third order interaction, *F* (1,76) = 0.05, *p* = 0.82, η^2^*_*p*_* = 0.00].

These results indicate that option choice was more difficult in the medical examination than in the travel package, and that younger adults felt more difficulty in choosing options than did older adults. Moreover, + OF and –OF did not differ significantly in terms of choice difficulty.

### Choice satisfaction

With regard to the choice satisfaction, the ANOVA showed a significant main effect of product [*F* (1,76) = 14.48, *p* < 0.001, η^2^*_*p*_* = 0.16]. The other main effects were not significant [age group, *F* (1,76) = 0.95, *p* < 0.33, η^2^*_*p*_* = 0.01; option framing, *F* (1,76) = 0.38, *p* = 0.43, η^2^*_*p*_* = 0.01]. The interaction between product and age group was significant [*F* (1,76) = 7.71, *p* = 0.01, η^2^*_*p*_* = 0.09], while the other interactions were not significant [product and option framing, *F* (1,76) = 0.26, *p* = 0.61, η^2^*_*p*_* = 0.00; age group and option framing, *F* (1,76) = 0.64, *p* = 0.43, η^2^*_*p*_* = 0.01, third order interaction, *F* (1,76) = 0.52, *p* = 0.47, η^2^*_*p*_* = 0.01]. A *post hoc* analysis for the interaction between product and age group showed that, when comparing satisfaction between age groups by product, older adults were significantly more satisfied with medical examinations than younger adults (*p* = 0.03). Moreover, for younger participants, satisfaction with the travel package was significantly higher than satisfaction with the medical examination (*p* < 0.001).

## Discussion

In this study, we used behavioral indices such as the number of options chosen, total price, choice difficulty, and choice satisfaction to 1) examine the effect of age on option framing and 2) the effect of different product types on the option framing effect based on two products: a travel package for enjoyment and a medical examination to identify a disease.

### Age difference in option framing

Previous studies on younger adults suggest that option framing is associated with loss aversion, where fewer options are deleted to avoid functional loss in –OF and fewer options are added to avoid monetary loss in + OF (Park and Kim, 2012; Herrmann et al., 2013). Levin et al. (2002) point out that in –OF, consumers tend to be hesitant to delete options due to the endowment effect, that is, the consumers value what they own more. Cognitive biases caused by the heuristic process such as loss aversion and endowment effects associated with option framing effects are less affected by aging (Kovalchik et al., 2005; Seaman et al., 2018). In addition, option choice involves a trade-off between higher quality and lower price. Such trade-offs require cognitive effort. Therefore, older adults with declining cognitive function may rely on more heuristic judgments and exhibit stronger option framing effects than younger adults (H1a). However, the results of the experiment showed that there was an option framing effect among older adults with declining cognitive function for both the travel package and the medical examination, as well as younger adults. In other words, hypothesis H1b (“There is no difference in the option framing effect between older and younger adults”) was supported. In decision making, older adults can supplement age-related declines in fluid intelligence, such as processing speed and working memory, with crystalline intelligence, such as previous experience and knowledge (Li et al., 2013). In option framing, Peng et al. (2016) reported no age-related differences since the rich experience of older adults compensates for age-related cognitive decline. The finding that younger adults had more choice difficulty than older adults for both + OF and –OF also supports this interpretation. In spite of age-related cognitive decline, older adults do not have difficulty executing function-price trade-offs that require cognitive effort. Although data on purchase experience were not obtained in this study, older adults, compared to younger adults, probably have more experience traveling and getting medical examinations. The option framing effect may not have been different from that of younger adults because older adults compensate for cognitive decline by previous experiences.

With regard to the hypothesis H1c, older adults show a positivity effect, that is, they pay more attention to and remember more positive rather than negative information and focus on positive information to make decisions (Fung and Carstensen, 2003; Mather and Carstensen, 2005; Notthoff and Carstensen, 2014). Positive aspects of + OF are the functional gains from adding options, while positive aspects of –OF are the monetary gains from removing options. Therefore, older adults, compared to younger adults, were expected to choose fewer options in –OF and more options in + OF (H1c). This hypothesis, however, was not supported. Positivity effect is driven by top-down processing (Reed and Carstensen, 2012), while option framing effect is driven by bottom-up processing, such as heuristics. Therefore, as the heuristics process was more dominant than the top-down process during option choice, H1c would not have been supported.

### Differences in option selection in products

We predicted that, in terms of a medical examination aimed at managing losses, the number of options chosen by older adults would not differ between + OF and –OF, and that older adults would choose more options than younger adults would in both framing conditions (H2a). The results partially supported this hypothesis. In medical examination, although the older adults showed the option framing effect as well as the younger adults, older adults chose more options for both –OF and + OF than younger adults. This age difference was not observed for the travel package. The differences in the age-related effects on the products may be reflective of the shift in goal orientation across adulthood. Younger adults reported a primary growth orientation in their goals, and loss prevention was more prevalent among older adults (Baltes, 1997; Ebner et al., 2006). In a product aimed to manage loss, such as a medical examination, enhanced functionality is positive information for older adults. Since older adults value emotional satisfaction (Carstensen, 2006), compared to younger adults, they have probably chosen more options for medical examinations.

In addition, older adults who value positive information have reported higher post-decision satisfaction (English and Carstensen, 2015). Therefore, regarding choice satisfaction, we hypothesized (H2b) that older adults would be more satisfied with their choice than younger adults, regardless of the product. The results demonstrated that this hypothesis was only valid for the medical examination. Since older adults are loss prevention-oriented (Baltes, 1997; Ebner et al., 2006), in medical examinations aimed at loss prevention, there is a possibility that the choice satisfaction of older adults was higher than that of younger adults. Meanwhile, younger adults chose fewer options, had greater choice difficulty, and had lower choice satisfaction than the older adults in medical examinations. The trade-off between function and price increases cognitive load, leading to negative affect and choice difficulty (Park and Kim, 2012). Younger adults, who have lower disease risk than older adults, attempted to choose the minimum options necessary, which may have increased choice difficulty and decreased choice satisfaction.

### Conclusion and limitations

The present study showed that the option framing effect was observed in both older and younger adults and suggested that older adults compensate for cognitive decline with their accumulated experience. These results suggest that it may be beneficial for sellers to present –OF options, regardless of the age of the consumer. Especially in the case of products related to managing losses—such as medical examination—older consumers not only chose more options, but also were more satisfied with their choices. From the consumer’s perspective, it is reasonable to assume that they would prefer making a choice without being influenced by option framing. Herrmann et al. (2013) suggest that in option choice, consumers are more likely to accept information from reliable sources. Therefore, they will be able to avoid the option framing effect by getting credible advice from reliable sources.

There are several points that must be considered in clarifying the effects of aging on option framing. Previous studies indicate that the mood state of older adults influences their decision making. Growney and Hess (2019), for example, report that positive moods may make it more likely for older adults to systematically process potentially important negative information, such as risk factors related to medication and treatment. It will be necessary to examine the effect of mood state on the decision trade-off between function and price at option choice. Older adults also prefer that fewer options are presented (Reed et al., 2008). Although 10 options were presented in this study, it is possible that the option selection strategies for older adults may be quite different between around five options that seem more manageable and 20 options that are difficult to manage. It is therefore necessary to examine the effect of aging on option framing, based on the number of options presented.

A limitation of this study is that the experiment was conducted in a laboratory environment, which greatly differs from a real-life situation. Moreover, the participants did not actually purchase products, and we did not measure attitudes toward the products, such as how much the participants felt they needed each product. However, a study on younger participants confirms the option framing effect in realistic situations (Herrmann et al., 2013), similar studies with older adults are required to confirm whether the results of this study can be generalized. Additionally, the sample size in this study was not large, 20 participants in each cell, and may not have been sufficient to detect an interaction between the option framing effect and age. It is, therefore, necessary to examine the effect of age on option framing with a larger sample size in the future.

## Data availability statement

The raw data supporting the conclusions of this article will be made available by the authors, without undue reservation.

## Ethics statement

The studies involving human participants were reviewed and approved by the Ethics Committee of the Graduate School of Human Development and Environment in Kobe University. The participants provided their written informed consent to participate in this study.

## Author contributions

KM and MT contributed the concept and design of the study and performed the statistical analyses. MT and KY collected data and controlled quality. MT collected the relevant literature and wrote the portions of initial drafts. KM drafted and edited the manuscript. All authors contributed to the article and approved the submitted version.

## Appendix

**TABLE A:** Screen for choosing the travel package options in +OF.

Please check the option if you do need it.			
Option	Description	Price	Check box
Landing on Phantom Island	An island made of sand that appears only when the tide is out. Walking around the island, you feel as if you are walking on the sea.	¥ 5400	□
Remote island experience	Mangrove cruise in the subtropical jungle of Iriomote Island and buffalo cart experience crossing to Yubu Island.	¥ 8600	□
Pay TV card	Pay TV at the hotel. Unlimited access to channels.	¥ 2000	□
Dinner	Luxurious dinner using local ingredients.	¥ 7000	□
Open air bath	Access to the hotel’s large public bath with open-air bath, as well as a free 30-min massage.	¥ 3500	□
Rent-a-car	Compact car.	¥ 12000	□
Airline ticket upgrade	Upgrade from economy class to first class.	¥ 9000	□
Hotel upgrade	Rooms can be changed from no room assignment to a room with a view facing the ocean.	¥ 6600	□
Insurance	Compensation for medical expenses for illness or injury while traveling, and for theft.	¥ 1200	□
Breakfast	Hotel buffet breakfast.	¥ 3200	□

**TABLE B:** Screen for choosing the medical examination options in -OF.

Please uncheck the option if you do not need it.					
Option	Description	Price	Check box
Gastroscopy	It is better than a gastric x-ray to check for esophageal, gastric, and duodenal cancer.	¥ 5400	
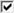
Test for sleep apnea	Wearing sensors on the fingers and under the nose to check for apnea, the state of breathing during sleep	¥ 8600	
Visceral fat CT-scan	The CT-scan measures visceral and subcutaneous fat to assess the degree of obesity.	¥ 2000	
Allergy test	Blood tests are used to determine the presence and degree of typical allergies such as cedar pollen, grass pollen, house dust, etc.	¥ 7000	
Thyroid function test	Thyroid function tests are performed to evaluate the status of thyroid function by measuring hormones (TSH, Free T_4_, and Free T_3_) in the blood.	¥ 3500	
Lung CT-scan	A CT-scan is used to take cross-sectional images of the lungs to investigate diseases such as early-stage lung cancer and emphysema.	¥ 12000	
Echocardiography	An echocardiogram (echo) is a test that uses ultrasound to visualize the heart to determine its size, movement, valve status, and blood flow.	¥ 9000	
Tumor marker diagnosis	The risk of cancer of the liver, colon, gall bladder, and pancreas is tested by cancer-specific substances in the blood.	¥ 6600	
F undus examination	A fundus camera is used to examine the blood vessels, retina, and optic nerve in the fundus.	¥ 1200	
Mental health	A questionnaire-based test assesses mental health status.	¥ 3200	
